# Focusing on Coal Workers’ Lung Diseases: A Comparative Analysis of China, Australia, and the United States

**DOI:** 10.3390/ijerph15112565

**Published:** 2018-11-16

**Authors:** Shuai Han, Hong Chen, Maggie-Anne Harvey, Eric Stemn, David Cliff

**Affiliations:** 1 School of Management, China University of Mining and Technology, Xuzhou 221116, Jiangsu, China; hessa1222@163.com; 2 Sustainable Minerals Institute, The University of Queensland, Brisbane 4072, Australia; m.harvey@uq.net.au (M.-A.H.); e.stemn@uq.edu.au (E.S.); d.cliff@mishc.uq.edu.au (D.C.); 3 Environmental & Safety Engineering Department, University of Mines and Technology, Tarkwa, Box 237, Ghana

**Keywords:** lung diseases, coal dust, monitor, compensation, governance, public health

## Abstract

China has high and increasing annual rates of occupational lung diseases such as pneumoconiosis and silicosis. In contrast, Australia and the United States of America (USA) have greatly lowered their annual rates of lung diseases since the 1970s. This paper systematically compared and analysed the multi-elements of coal dust management and health management in these three countries to provide a reference for China. Regarding coal dust management, this paper found that coal workers in China are more susceptible to lung diseases compared to workers in the USA and Australia, considering fundamental aspects such as mine type, coal rank, and geological conditions. In addition, the controllable aspects such as advanced mitigation, monitoring methods, and the personal protective equipment of coal dust were relatively inadequate in China compared to the USA and Australia. Health management in China was found to have multiple deficiencies in health examination, co-governance, and compensations for coal workers suffering from lung diseases and healthcare for retired coal workers. These deficiencies may be attributed to insufficient medical resources, the Chinese government-dominated governance, ineffective procedures for obtaining compensation, and the lack of effective and preventive healthcare programs for the retired coal workers. Based on the USA and Australia experience, some suggestions for improvement were proposed.

## 1. Introduction

Coal resources are an important component of the world energy supply, accounting for 27.62% of primary energy consumption worldwide [1,2]. This is particularly true for China, where coal accounts for more than 60% of the national energy consumption [3]. With the development and utilisation of coal resources in the past and present, the legacy of health problems (lung diseases) is increasingly apparent. These health problems develop from the creation of dust particles in the coal production process and the reaction of the lung tissue to the dust, namely pneumoconiosis [4] (Coal workers’ pneumoconiosis (CWP) is a broad group of pneumoconiosis caused by exposure to respirable coal mine dust over several years. Dust particles of less than five microns can penetrate to the peripheral bronchioles and alveoli, where they can block air passages and cause primary lesions containing coal dust, macrophages, and fibroblasts. If free silica is included in the dust, then fibrogenic substances can be released. Two forms of CWP can be distinguished. The first is a simple form where coal macules are surrounded by fibrosis, or small scars of less than 10 mm in the lung. Symptoms can include a cough or shortness of breath; however, sometimes, there may be no symptoms at all. The other form is the complicated form, or progressive massive fibrosis (PMF). PMF is associated with fibrosis or scarring in the lung of 10 mm or greater. Symptoms include shortness of breath, black sputum, chronic cough, pulmonary hypertension, frequent pneumonia, and heart problems). It was estimated that 25,000 and 46,000 coal worker’s deaths were caused by pneumoconiosis (CWP and black lung, respectively) and silicosis globally in 2013 [5]. Silicosis, asbestosis, and pneumoconiosis accounted for 29,000 deaths worldwide in 2009 due to exposure to silica, asbestos, and coal dust in developing countries [6]. The Global Silicosis Elimination Plan in the World by 2030 has been promoted by the WHO (World Health Organisation) and the ILO (International Labour Organisation) to reduce the impact of these diseases [7]; however, this still remains a significant challenge in China [8]. According to the latest reports in China, there were 27,992 new cases of pneumoconiosis reported, making up 88.06% of the 31,789 cases of occupational diseases reported in 2016, and increasing by 1191 cases compared to last year [9]. Coal miners’ pneumoconiosis (CWP) and silicosis accounted for 95.49% of the pneumoconiosis reported, with 16,658 and 10,072 cases reported in 2016, respectively. The total number of pneumoconiosis cases reached 72,000 for workers up until 2015, with 6000 deaths occurring per year [10]. In fact, Chinese officials have acknowledged that the reported data is only based on diagnosed cases, and there is likely a high number of unreported and undiagnosed cases [8]. A huge gap exists between China’s energy and economic development and the lives and health of Chinese coal miners. Meanwhile, health management is lagging behind safety management in China, as the accidental death rate per million tons of coal reduced from 5.07 to 0.156 between 2001–2015, despite the rise in occupational pneumoconiosis cases [11]. Both the government and coal mines put more effort and money into safety rather than health based on the trade-offs between “the visible and immediate accident” and “invisible and chronic lung diseases”. This seriously damages coal miners’ lives and health, and restricts the healthy development of the coal industry. There are currently few studies that systematically describe the extent of the problem in China [12].

China, the United States of America (USA), and Australia were the top three coal-producing countries in the world in 2017, in which the production of coal was (million tonnes oil equivalent) 1747.2 Mt, 371.3 Mt, and 297.4 Mt, respectively [2]. Additionally, China, the USA, and Australia also have some of the largest proved coal reserves in the world. However, the USA and Australia focused on the improvement of occupational health earlier than China, acknowledging it as important as a safety issue [13]. Compared to the prevalence of CWP in China (6.02% in Chinese coal mines; 9.86% in locally-owned mines, 4.83% in state-owned mines) [14], the USA and Australia are lower (in the USA from the 1990s to the 2000s, 3.2% of workers; from 2005 to 2014, 1.88% of workers [15], and in New South Wales (NSW) in Australia, the prevalence in workers was <0.5% [16]). Obviously, the USA and Australia are ahead in terms of CWP prevention compared to China. Furthermore, CWP and increasing silicosis attracts more public attention in these countries, and the USA and Australian governments have adopted countermeasures and responses [17]. Overall, the USA and Australia are not only the top coal-producing countries, they are also the countries with sound occupational health management systems and extensive governance experience for diseases caused by coal dust, which are worth using as a reference for China.

Previous studies have mostly taken a single perspective on coal dust control such as control technology and equipment, or the risk of coal dust or silica exposure [18,19], lacking the systemic considerations of coal workers’ lung diseases in various countries. This paper extends previous studies of coal workers’ lung diseases by comprehensively comparing the current statistics of coal workers’ lung diseases and the entire process of coal dust management and health assessment management within three major coal-producing countries. Coal dust management included coal dust sources (coal mine type and geological condition) and coal dust control (mitigation and monitoring methods of coal dust) within the three countries. In addition, the health assessment management focused on the discovery of lung diseases (periodic health examinations, standard of radiographs and health assessment methods), the governance of lung disease issues (pattern of governance and participants), and the treatment of lung diseases (insurance, compensation, penalty for violations, and healthcare for retired coal mine workers). For this paper, the data for China was collected from the website of the National Health and Family Planning Commission (NHFPC), which was formerly known as the Ministry of Health (MOH), the Chinese Center for Disease Control and Prevention (CCDCP) subordinated by the NHFPC and the National Bureau of Statistics of China (NBC). The data for Australia was collected from the Australian Bureau of Statistics (ABS), Coal Service (NSW), and Queensland Government (QLD). The data for the USA was mainly collected from the Bureau of Labor Statistics (BLS), Mine Safety and Health Administration (MSHA), the Occupational Safety and Health Administration (OSHA), the Center for Disease Control and Prevention (CDC), and The National Institute for Occupational Safety and Health (NIOSH).

## 2. The Situation of Coal Workers’ Lung Diseases in China, Australia, and the USA

### 2.1. Coal Production in China, Australia, and the USA

**Figure 1:**
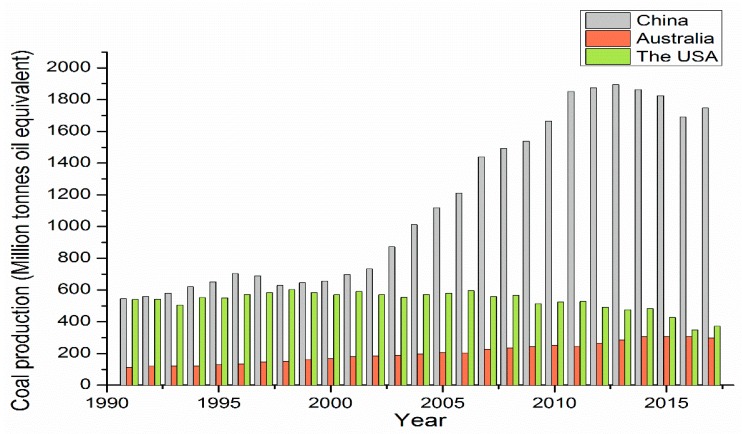
The total annual coal production (Mt) in China, the United States of America (USA), and Australia from 1990 to 2017. Data from 1990 to 2017 comes from a British Petroleum energy review.

Nowadays, China occupies a principal position in the world’s coal production: coal production reached 1747.2 Mt (Million tonnes oil equivalent) in 2017 [2], which was 4.7 times the coal production in Australia, and 5.8 times that of the USA (Figure 1). It suggests a higher possibility of respiratory diseases in China because of the higher volume of coal dust produced during coal processing.

Coal production has been rapidly increasing since 2000 in China, as indicated by the rapid growth of coal mining enterprises. More specifically, the coal production of China steadily increased from 1980 to 1995, where a large number of small-scale coal mines (small local-stated, township, village, and private coal mines) emerged (Figure 1). These accounted for nearly 73% of all Chinese coal mines, and resulted in a huge number of fatalities. Consequently, Chinese coal was called “blood coal” during that period [20]. After that, the Chinese government became aware of the problem, and started the closure of small-scale coal mines in 1993, resulting in a decrease of small coal mines and coal production until 2000 [21]. However, with rising demand, economic development, and the increased strength of resource consolidation for coal mines, coal production was continually rising, and the numbers of small coal mines were declining. Nonetheless, a large number of small coal mines still existed, and it was estimated that there were 9690 townships, 1666 locally-owned coal mines, and 1493 key state-owned coal mines in 2012 [22]. In 2017, the coal mines with a production capacity of less than 300,000 tons accounted for 25.4% of all coal mines, and coal mines with a production capacity between 300,000–1,000,000 tons accounted for 55.9% of all coal mines [23]. Overall, the current state of Chinese coal mines is the continued co-existence of the key and large stated-own coal mines and small coal mines in China [24]. In Australia, there is a steady increase in coal production, but the increasing range is smaller than that of China (Figure 1). Notably, there are no stated-owned coal companies in Australia compared to China, and the ownership of coal mines is attributed to private and large-scale coal operations, including BHP Billiton Pty Ltd., Peabody Pacific Pty Ltd., and so on [25,26,27,28]. As for the USA, coal production is in decline; it is being gradually replaced by newer energy resources. Again, large non-state coal operations dominate the ownership of coal mines, including Peabody Energy and Arch Coal [2,27,29].

### 2.2. Coal Miners in China, Australia, and the USA

**Figure 2:**
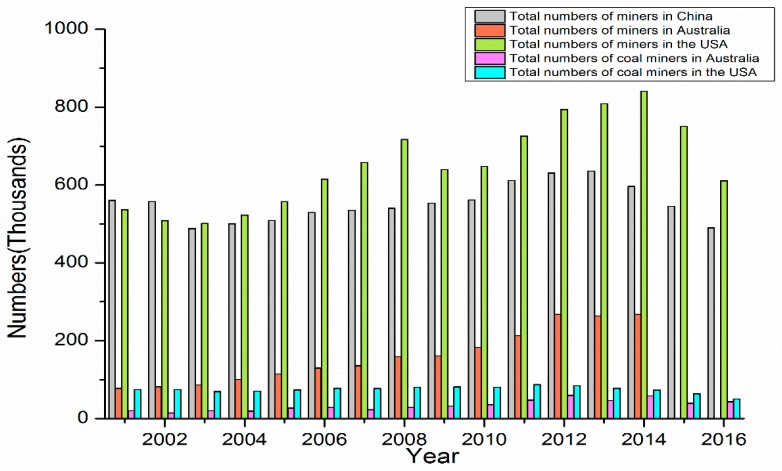
Total numbers of miners and coal miners in China, Australia, and the USA. Data from 2002 to 2016 comes from the websites of the National Bureau of Statistics of China, Australian Bureau of Statistics, and Bureau of Labor Statistics of the USA, and the National Institute for Occupational Safety and Health.

Not only is there higher coal production in China compared to the USA and Australia, but the number of coal mine workers is also higher (Figure 2). Consequently, more coal workers in China will be exposed to the hazards of coal dust, leading to a higher likelihood of contracting lung diseases.

Figure 2 showed that the numbers of miners in China and the USA are far higher than those in Australia. In 2016, there were 43,700 coal miners in Australia and 50,680 in the USA [30,31,32,33]. In China, there is lack of distinction between the various sectors of the mining industry, such as coal mining and metalliferous mining. Due to this, the number of miners is used as a proxy for the number of coal miners [34]. However, there was a report coordinated by the Division of Coal Bureau and the State Administration of Work Safety (SAWS) in 2013 on the labour management of coal mine workers in China, which reported 525,000 coal miners (the total number of miners in China was 631,000), 304,000 of which were underground coal miners. The number of coal miners in the key state-owned, locally state-owned, and township coal mines were 222,520, 68,910, and 134,550, respectively [22]. Comparatively, the number of coal miners in the small coal mines was much higher than all of the coal miners in the USA and Australia.

### 2.3. The Pneumoconiosis of Coal Miners in China, Australia, and the USA

In China, there were 21,719 new cases of occupational diseases reported per year from 2003 to 2016 on average (Table 1; [35,36,37,38,39,40,41,42,43,44,45,46,47]). Therefore, new cases of lung diseases caused by coal dust made up 44.83% of all occupational diseases from 2003 to 2016. Furthermore, coal workers’ pneumoconiosis and silicosis accounted for 94.39% of all pneumoconiosis from 2009 to 2016. Since 2010, more than 20,000 cases of coal workers’ pneumoconiosis and silicosis have emerged per year, and there was an average of 723 deaths per year from 2007–2011. Post-2011, the data for mortality from pneumoconiosis in coal workers is unavailable from the NHFPC. Thus, the existing statistics are likely an underestimation, due to the lack of diagnosis and mortality attribution.

**Figure 3:**
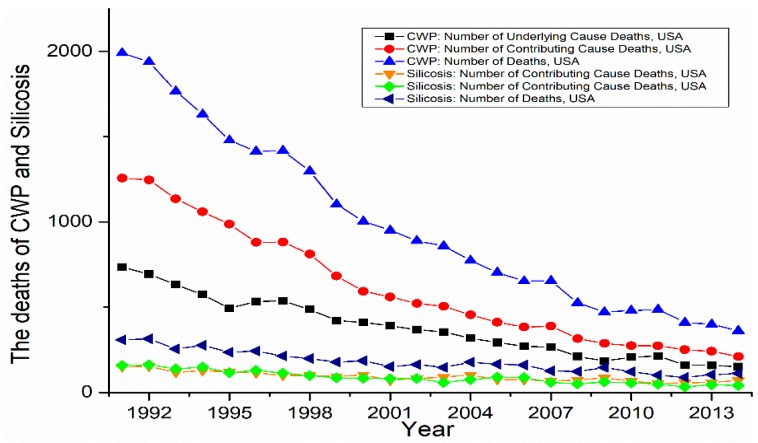
The deaths of CWP and silicosis in the USA from 1992 to 2013. Data comes from the Center for Disease Control report (Work-Related Lung Disease Surveillance Report, 2007) and the National Institute for Occupational Safety and Health website.

In the USA, there were 37,965 cases of confirmed CWP (International Labour Office (ILO) category) reported by the NIOSH from 1968 to 2015 [47]. Furthermore, the number of deaths of coal miners with CWP as an underlying or contributing cause was more than 75,000 since 1968 [48]. Overall, the deaths of coal workers pneumoconiosis and silicosis gradually decreased from 1990 to 2014 (Figure 3) [48]. There was a cluster of 60 cases of PMF diagnosed in current and former coal miners in an eastern Kentucky radiology practice in 2015–2016 [49], which accounted for a resurgence of pneumoconiosis and silicosis [50].

**Figure 4:**
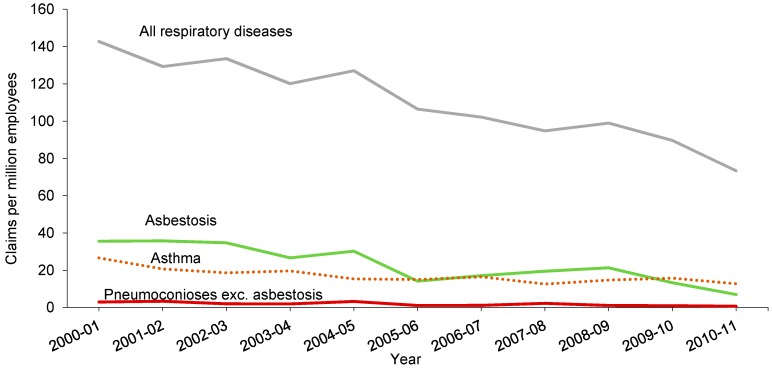
The claims of respiratory diseases per million employees from 2000 to 2011. Data comes from the report of Occupational Respiratory Diseases in Australia.

In Australia, very few cases of CWP were confirmed by government agencies (Figure 4) [51]. A 24-year mortality surveillance study reported that while there were over 1000 pneumoconiosis-related fatalities in Australia between 1979–2002, CWP accounted for fewer than 100 fatalities, with the steepest decline of deaths occurring between 1988–1996 [52,53]. Meanwhile, there were fewer than five cases per million employees for pneumoconiosis (excluding asbestosis) from 2000 to 2008, and no claims from 2008 to 2011 (Figure 4) [54]. The Queensland government reported only 26 cases of CWP and nine cases of silicosis from 1986 to 2018. “Black lung” as a potentially fatal respiratory disease had been regarded as eradicated from the Australian coal industry until new cases emerged in 2015 in Queensland. Since May 2015, 21 current and former coal mine workers in Queensland have been diagnosed with CWP [55]. Furthermore, one new case was found in NSW in 2017, which was the first since 1970 [55]. This is a cause for concern for the Australian government and the mining industry. This has resulted in a wholesale overhaul of the health surveillance system in Queensland.

The different standards for reporting and diagnosis, as well as non-public statistics of pneumoconiosis between countries hindered the direct comparison of the deaths, cases, and prevalence of pneumoconiosis for coal workers per year. However, it was obvious that the pneumoconiosis of coal miners in China was more serious than that in Australia and the USA. Despite the slight increase in pneumoconiosis cases for coal miners of the USA and Australia in recent years, the overall trend of pneumoconiosis cases for coal miners is a gradual decline over the long term. Historically, the USA and Australia had passed the epidemic threshold level for CWP during the 1968–1977 period, and have more recently entered a relatively low and stable phase [56]. However, the rapid development of coal mines in the USA was 70 years earlier than their development in China, which may indicate that Chinese pneumoconiosis cases have not reached an epidemic threshold level, and are rapidly spreading due to the progress and development of the coal industry. The epidemic threshold level of CWP will come soon in China, because the length of time that it takes for coal workers to contract CWP shortens over time, with a median length of service of 20 years [57]. Based on these predictions, China may soon be facing a period of higher deaths from invisible causes such as pneumoconiosis, compared to immediate accidental deaths [58].

## 3. Comparative Analysis of China, Australia, and the USA

### 3.1. The Coal Dust Management

There is no specific treatment for CWP or other diseases caused by coal dust, and the best and most effective protection from harm is through elimination by preventing the initial exposure to coal dust [59]. While the hazard itself cannot be completely eradicated because of the structural geology and coal rank, it can be controlled and eliminated by applying engineering controls, as well as monitoring respirable coal dust and personal protective equipment. Comparisons of these factors between China, Australia, and the USA are listed in Table 2.

#### 3.1.1. The Geological Conditions of Coal Mine

Many factors of coal extraction and coal dust management illustrate the fundamental weaknesses of Chinese mining practices compared with the USA and Australia (Table 2). First, 94.3% of all Chinese coal mines are underground mines (*N* = 7723) and open-cut mines (surface mines) only accounted for 5.7% (*N* = 439) [60]. In contrast, the USA and Australia (Queensland, QLD and NSW) have a higher percentage of open-cut coal mines (surface mines), 72.26% and 64.13% (QLD: 74%; NSW: 52.38%), respectively [61,62,63]. Underground coal miners are at greater risk of developing CWP than strip or surface miners because of the denser dust concentration in the underground environment due to the confined ventilation circuits of the underground mine, while the coal dust can be diluted by ambient air for surface mines [64]. Increased exposure to this cumulative dust increases mortality from lung cancer [65]. Additionally, the structural geology of China combines complex folds and faults with thin–medium width coal seams, which increases the collision of the rock during the mining and extraction process, and the soft nature of the host rock exacerbates coal dust production.

The coal rank is the progressive alteration (coalification) from lignite (lowest) to anthracite (highest). It has been noted that higher rankings of coal will cause a greater prevalence of all of the categories of pneumoconiosis [66,67]. By comparing the coal rank of reserve coal in the three countries, it was found that the proportion of lignite was higher in Australia than the USA and China [16,68], while China has a higher proportion of anthracite than the USA, making Chinese coal more dangerous for health. Regardless, all three countries are currently encountering a new challenge that comes with the progressive mechanization of mining processes; more quartz dust is being generated through activities such as cutting or drilling rock. Associated silica exposure, which is more dangerous than coal dust as it causes complicated pneumoconiosis, was implicated in the rise of CWP and silicosis in USA and Australia recently [59,69]. Besides that, coal workers were developing more complicated combinations of diseases such as Caplan’s syndrome, which is an association of rheumatoid arthritis with coal pneumoconiosis and progression into severe fibrosis. It was estimated that 2–3% of coal miners have Caplan’s syndrome, and as such, the disease should be given the attention and resources it deserves.

#### 3.1.2. The Mitigation of Coal Dust

The mitigation of coal dust through engineering controls is a direct and effective way to manage hazard sources. These engineering controls are similar in the three countries in that they utilise techniques such as ventilation, coal seam water infusion, water sprays, automated and remote equipment. The more advanced mitigation equipment, especially for automation and remote equipment operation, are generally used in large coal mine companies compared to the smaller coal mines in China [70]. In the locally-owned and village coal mines, updates to equipment were slow, and much of it was old and ineffective [71]. Although the key state-owned coal mines were able to update the equipment in a timely manner for the reduction of coal dust, China still lagged behind the USA in levels of automation, efficiency, and cost. Presently, the USA attaches more importance to the technological development of coal dust control. Institutions such as the Pittsburgh Research Laboratory and the Spokane Research Laboratory, as well as National Personal Protective Technology Laboratory under the NIOSH, are responsible for the newest research and development of dust control. For example, Pittsburgh Research Laboratory features a full-scale above-ground longwall coal mining laboratory to develop and test new dust mitigation methods and technologies [55,72]. Additionally, Pittsburgh Research Laboratory also has a full-scale continuous miner dust laboratory, where the scientists can test technologies to control respirable dust with parameters such as water spray, face ventilation, mining height, and the automation of machine position [55].

Personal protective equipment such as respiratory protective equipment can work in conjuncture with engineering controls to minimise and control respiratory exposures, but are also ultimately the last line of defence on coal dust prevention. Although the kinds of filtering respiratory protective equipment were the same in all three countries, the standard and usage rate was different. The standard of respiratory protective equipment in the USA was much stricter than the other countries, including the N95\N99\N100 (not resistant to oil), R95\R99\R100 (resistant to oil), and P95\P99\P100 (oil proof) [73,74]. Australia adopted P1 (mechanically generated particulates, i.e., silica, asbestos), P2 (mechanically and thermally generated particulates, i.e., metal fumes), and P3 (highly toxic materials such as beryllium) standards, which were consistent with the European standards [75]. In China, there were two kinds of respirators: KN90\KN95\KN100 (not resistant to oil) and KP90\KP95\KP100 (resistant to oil) [76].

The NIOSH required the N95 (95% filter efficiency) mask as the least depending on the workers’ exposure to different types of coal dust and crystalline silica [74]. However, in China, field investigations suggested that this requirement is not adequately guaranteed for coal workers’ respiratory protective masks, especially in small coal mines. In addition, the quality of the masks that were used in the USA and Australia in terms of material, air resistance, comfort, and dust volume were better than that in China [76].

#### 3.1.3. The Monitoring of Respirable Coal Dust

At present, the occupational exposure limit (OEL) is the general concept of coal dust exposure concentration, where many countries adopt the standard of threshold limit value (TLV) replacing the prior standard of maximum allowable concentration (MAC) [77]. The MAC is the highest ceiling concentration, whereas the TLV is a time-weighted average of the concentration of the hazardous agent in the atmosphere. TLV included the time-weighted average (TLV-TWA) and short-term exposure limit (TLV-STEL). The USA and Australia adopted the TLV-TWA, and China transferred the MAC to the TLV-TWA after 2004, in which the implemented system follows the current standard of the personal exposure concentration of respirable coal dust in the air of the workplace (AQ 4202-2008) [78]. Although all three countries adopted the TLV-TWA standard, there were different values for the OEL in China, Australia, and the USA (Table 2). In China, the OEL is characterised as the respiratory dust limit (RDL), which in turn is based on the different concentrations of free silica (CFS). When the CFS was less than 5%, the RDL was 5.0 mg/m^3^, and the CFS was 5–10%; then, the RDL was 2.5 mg/m^3^, and so forth (Table 2). In the USA, the RDL was reduced from 2.0 mg/m^3^ to 1.5 mg/m^3^ for underground mines, when the CFS was more than 5% since 2014 [79]. Additionally, the MSHA applied other exposure limits in August 2016; the concentration limits for safe respirable coal mine dust for intake air reduced from 1.0 mg/m^3^ to 0.5 mg/m^3^ for coal miners who have developed pneumoconiosis [80]. The total coal dust concentrations were 3.0 mg/m^3^ in Queensland and 2.5 mg/m^3^ in NSW, and regarding the adjusted RDL on the basis of 2.2 L/min in the AS 2985 method and 2.0 L/min in the MSHA, the RDL in Queensland and NSW were 3.7 mg/m^3^ and 3.1 mg/m^3^, respectively [16]. Compared with China and Australia, the standard of RDL in the USA is stricter. In reality, the concentration limits were not firmly implemented in China, and it was estimated that the maximum concentration of coal dust was between the 198–3420 mg/m^3^ from 1983 to 2008, which was 49.5–855 times that of the standard of occupational exposure limit [81].

Monitoring devices and methods are necessary tools supporting the control and testing of respirable coal dust. There are three categories of devices for monitoring: gravimetric sampling devices, static monitoring devices, and real-time personal dust monitoring devices (including light scattering (laser photometry) devices and tapered element oscillating microbalance (TEOM) devices) [55]. Gravimetric sampling devices are used for personal samples or designated operator sampling, which is the main and most basic sampling method in the USA and Australia. In addition to personal sampling, area sampling is also commonly used in Chinese coal mines. However, area sampling is usually done for only 15 minutes, which can give inaccurate results [82]. For monitoring samples, the methods of determining the maximum risk subgroup and similar exposure group (SEG) were similar in the USA, China, and Australia. However, the sample size in the USA is larger than it was in China, for example when the number of the SEG in the USA was 15, the sample size in China was eight [83]. 

Australia is adopting the “single-sample” method, which was evaluated by comparing each dust sample individually to the applicable dust standard. Before 2014, the USA always used the “multiple samples” method, but the final rule (FR 75 64412) authorized the MSHA to cite an operator based on a single MSHA sample showing excessive dust, rather than on an average of multiple samples [84]. Currently, China still uses the maximum value of all of the samples in accordance with the occupational exposure limit standard. Furthermore, there is an absence of systems managing statistics and re-detection as well as evaluation of the occupational exposure limit [84]. The periodic monitoring of dust was similar between China and Australia, as mine operators were required to conduct sampling at least once every two or three months [55,85]. Notably, in NSW, the personal sample test was generally carried out by a third party (coal service), but in China, this testing was completed by coal mine itself, and was reported to the local department of safety and health, which resulted in several false reports or the concealment of data [86]. In September 2008, coal mines began using real-time personal dust monitors (PDM), utilising the gravimetric methods (PDM3700) in underground coal mines [13]. However, the usage of real-time monitoring was always inadequate in China, even in the key state-owned coal mines, with the majority of real-time monitoring being light scattering [87]. This technique does not directly report actual dust concentrations, because the measurements are calculated by the size of the particles rather than their weight, which can easily be affected by environmental changes such as a lack of underground light [55,88]. Hence, even in the key state-owned coal mines, the coal dust exposure levels in tunnelling, mining, and combining areas were much higher than the occupational exposure limits [89,90,91]. The real-time TEOM device had been used in NSW, and Queensland has been promoting it in recent years. In addition, there has been concern that the failure of dust control may be related to the size and type of mining operations, as workers at some smaller operations and sub-contracted mines may not be adequately protected [92]. The survey from the LSP (Love Save Pneumoconiosis, a non-governmental organisation) based on 2277 migrant coal workers of CWP showed that only 5.9% of migrant workers were provided with respiratory protective equipment, 6.83% were educated by their employers about the hazards of coal dust, and 7.33% of coal mines were monitored for use of respiratory protective equipment [92]. The lack of respiratory protective equipment inspections also indicated that general coal mine inspections for coal dust management were inadequate. Zhu and Chen pointed out that the inspectors usually merely looked around without auditing or inspecting potential workplace hazards. Likewise, bribes were likely to occur as the inspectors would inspect the same coal mines over the long term [93]. Furthermore, mitigation and monitoring equipment were only used when the inspectors were on site.

### 3.2. The Health Management of Coal Workers’ Lung Diseases

Health management systems play an important role in reducing the impact of lung diseases on coal workers. Health management includes health assessment examinations, the governance of lung diseases by stakeholders, and insurance and compensation. Comparisons of the factors of health management in China, Australia, and the USA are listed in Table 3.

#### 3.2.1. The Health Examination for Coal Workers

Periodic health examination was a leading method of lung disease diagnosis in all three countries. However, the specific time period between health examinations was different in China, the USA, and Australia. While five years was an achievable goal for every country, China had periodical health examinations every two to three years based on the Coal Mine Safety Regulations by the State Administration of Coal Mine Safety [85]. This is more frequent than the health examinations in the USA and Australia. Although the period between health examinations was shorter than others, the accessibility of health examinations for coal workers was inadequate and extremely low [8]. This is especially true for small coal mines, where a lot of coal miners did not have any examinations during and after work. It was estimated by LSP that 84.6% of CWP cases come from small coal mines (of 2270 cases nationally). In addition to this, many village coal workers and their families would not attribute deaths to coal dust-related lung diseases due to the lack of health examinations or knowledge [94]. Even when workers are aware of the diseases, many coal workers decide to forgo treatment because of the high medical costs and the burden on their families [94]. Overall, the health examinations cannot be effectively guaranteed for all coal workers across China.

The regular examinations include chest radiographs (X-ray), respiratory assessment questionnaires, and spirometry testing. In China, occupational examinations and the diagnosis of occupational diseases must be processed in specific institutions. It was reported by the NHFPC in 2017 that there was a total of 2754 occupational examination institutions (0.2791% of all medical institutions) and 478 occupational diagnosis institutions (0.0484% of medical institutions) [95]. Meanwhile, there were only 543 medical practitioners (364 with a professional certificate) in all of the occupational examination institutions, and 117 medical practitioners (47 with professional certificate) in all of the diagnosis institutions in 2013 [96,97]. Prior to November 2017, diagnoses were completed and signed by three separate medical practitioners; however, this was abolished, as it may have been unnecessarily excessive in China [97]. In the USA and Australia, examination and diagnosis are often concurrent, and most of the medical institutions in these countries are qualified for occupational disease diagnosis. In addition, the NIOSH provides free mobile screening services to raise the accessibility of health examinations for coal workers. These are mobile units that travel across the USA, visiting coal mines and mining communities, even retirement communities, with notifications of the units’ locations available online six months in advance [98]. In comparison, the medical examination and occupational diseases identification services in China are substandard [99].

The USA and Australia are based on the same international classification of radiographs of pneumoconiosis from the ILO (negative, simple pneumoconiosis 1/2/3, complicated pneumoconiosis A/B/C). However, China uses a national diagnosis criteria for pneumoconiosis (GBZ70-2009, stage I, II, or III) instead of the ILO standard [90,100]. In addition, the NIOSH appoints different B readers (each with professional certificates, re-assessment examination every four years, and training courses provided by the NIOSH that are freely available online) to examine the radiographs. When two readers disagree on a diagnosis, the NIOSH will send the radiographs to a panel of three B readers to assess the case for CWP [101]. In Australia, the diagnosis of radiographs is generally conducted by two nominated medical advisors. Nominated medical advisors play an important role in the diagnosis of pneumoconiosis; previously, these nominated medical advisors were appointed by the coal companies, but recently, the government transferred their nomination to the commissioner for mine safety and health rather than the companies themselves to avoid potential conflicts of interest, changing the “Nominated Medical Advisor” to the “Approved Medical Advisors” [55]. Since the resurgence in CWP cases, there are now two highly qualified and proficient NIOSH certified B-readers in Australia; prior to March 2017, there were none [55]. At the same time, the USA was conducting a dual-read process, where every coal mine worker’s chest X-ray was examined twice: first by an Australian radiologist, and then by the USA-based NIOSH-approved readers [102].

#### 3.2.2. The Participants of Health Management

Cooperative governance was a basic method for solving the occupational problems in the USA and Australia, and this governance came from many sources, i.e., the government, coal mine companies, unions, non-governmental organisations, and the community [103,104]. Unions, which have the ability to hold the coal mine companies or governments to implementing health and safety laws, are largely absent from the governance of occupational health management in the Chinese system, in comparison to Australia and the USA [13,55]. For example, in Australia, the union was able to appoint the Industry Safety and Health representatives, who carry out inspections and investigations, advise on shortcomings, and report to the inspectorate or appeal to the committee if they are not satisfied with the actions of a site senior executive to rectify an issue. These representatives are also members of the statutory committee and the Standing Dust Committee, with representatives from the industry, unions, and government, and have the right to protect coal workers’ health against the industry stakeholders, even if that stakeholder is the government [55,105,106]. In addition, the government holds a hearing on occupational issues in the industry to identify solutions and share knowledge on emerging research and methods of dust suppression among unions, industries, and others parties [56].

In China, occupational health management was government-dominated [107,108]. The government is in charge of occupational health and safety management, and controls the investment of funds, the formulation of policies, the implementation of measures, and even the aftermath of accidents. However, other parties are obviously vulnerable to health issues, and they often do not take part in the governance of these health issues [109].

Firstly, enterprises are often passive, tend to avoid responsibility for occupational health and safety, are driven by short-term interests, and thus minimise investment in health and safety and business expenses [34];

Secondly, third-party (union) and non-governmental organisations are often unwilling to participate in the occupational health and safety problems that they are closely involved with. Subsequently, they seem to be inconsequential organisations that have no right to stand against the coal mines or the government. In fact, most injured workers mistrust trade unions because of corruption; sometimes, they side with employers [94].

Thirdly, the coal workers were mostly farmers and lacked the knowledge of their legal rights, i.e., the prevention of dust, the process of examination, their labour contract, and financial compensation [94].

Finally, the government-dominated management easily favoured particular interest groups, reducing the effectiveness of governance. Furthermore, the relevant responsibilities were always delegated to different departments of government, resulting in a low efficiency of governance. In 2010, the Occupational Safety and Health Supervision Division was officially transferred to the State Administration of Work Safety from the NHFPC in parallel with the State Administration of Coal Mine Safety [110], but the function of occupational health management was not yet fully clarified with the Ministry of Health. The transfer indicated an official linkage between safety and health management in China; however, this was decades behind similar moves made by the USA. Since the Coal Act (1969) and the Mine Act (1770), the NIOSH and Mine Safety and Health Administration are the two main departments that oversee health and safety responsibilities. The NIOSH was given the legislative responsibility to develop research into safe work practices, which would then inform the health and safety standards of the OSHA and MSHA, although the recommendations from the research outcomes are not legally binding [13]. In addition, there are conflicting or inconsistent policies and regulations between central and local government, and the coal mine companies and local government were often financially tied in China [24]. This direct governmental management of state-owned coal companies can lead to concealing and exonerating supervisors or coal operators that are implicated in health and safety violations. In contrast, most of the coal mines are private in Australia and the USA, so their governments are able to oversee health and safety standards in a relatively objective fashion. In addition, the national policies for the USA and Australia state that the penalty for operators who knowingly conceal cases of occupational disease will be liable for imprisonment, but for China, the same penalty is only applied in cases of serious coal mine accidents, not health issues.

#### 3.2.3. The Compensations of Coal Workers with Lung Diseases

Australia is a country where social welfare and social security are relatively sound systems, and occupational disease compensation can be efficiently implemented. Compared with China and the USA, WorkCover was the highest financial compensation, which was up to $325.70 weekly as of 1 April 2018 [111,112]. In the USA, the Division of Coal Mine Workers’ Compensation set up benefit rates (Part B and Part C) for lung diseases paralleling the WorkCover in the states, where the two parts of compensation ranged from $660.10 to $1320 monthly based on the number of dependent persons [113]. The compensation of Part C came from the Black Lung Disability Trust Fund, which is from the excise tax on sold coal, and is paid by the coal operator. Similarly, in Australia, some funds coving compensation come from coal industry operators such as the mining safety fund in NSW and the victim funds from industry levies [55]. However, in China, the compensation for coal workers was basically WorkCover from the Department of Social Security. The requirements for compensation include relevant materials such as the lung disease identification results and official diagnosis from the occupational diagnosis institutions, the assessment of labour ability from the labour bureau, and the labour contract from the coal mine operators, amongst other forms of paperwork. However, the application for compensation has to be submitted by the coal mining company rather than the worker themselves [114]. Furthermore, the treatment and reimbursement process for coal workers is usually derailed because of restrictive delays, arbitrary legal requirements and objections from employers, and is compounded by intransigence on the part of the local government [8]. Many coal workers (mostly migrant workers) were not eligible for compensation due to a lack of contracts or the premature termination of labour contracts by the coal mines in China [108]. The Chinese LSP reported that 90.5% of migrant workers with CWP have no formal labour contract, based on data collected from 2217 coal workers with CWP [93]. In 2016, an advance compensation payment was made for the “Interpretation on the improvement of prevention and treatment for migrant workers with pneumoconiosis” by the NHFPC (MOH), which was paid for by the social security department for the coal workers with informal labour agreements, in spite of the lack of contracts [115]. However, the informal labour agreements involved many lengthy legal proceedings, during which many coal miners gave up the fight for compensation. This type of advance payment did not work efficiently until now due to the convoluted bureaucracy of government departments [8]. Moreover, there is no compensation for coal workers who are unable to provide evidence of the informal labour agreement. Notably, in the USA, claimants and representative payees who are entitled to benefits are required to report any circumstances that could affect their entitlement to benefits or the number of benefits received to the Office of Workers’ Compensation Programs [116], but this feedback is virtually non-existent in China.

Retired coal workers enjoy the same welfare and treatment in the USA and Australia, that is, free and voluntary health assessment and compensation as a result of occupational dust, and even the evaluation, treating, counselling, and rehabilitation services of industry clinics or unions. However, in China, there are very few services for retired coal workers, especially for those without labour contracts. At present, approximately 1.3 million people are estimated to be at risk of unemployment (29.3% of China’s 4.44 million coal mining employees) because of the current reorganisation and closure of Chinese coal mine enterprises [117]. It would be easy to ignore health examinations and subsequent compensation for coal workers with forthcoming unemployment.

## 4. Conclusions and Suggestions

The following conclusions were made based on the multi-perspective analysis presented in this paper:

- China is the primary coal-producing country in the world and also has the largest number of coal workers, which is more than five times the number of workers in the USA and Australia. This means that more people are at risk of exposure to coal dust. At the same time, the Chinese coal mines were characterised as consisting of more small and state-owned mines compared with the large, private coal mines in the USA and Australia. These small mines are reporting more serious diseases due to the absence of lung disease management.
- The number of cases of lung disease in coal workers is rising every year in China; more than 20,000 new cases of CWP and silicosis have been appearing per year since 2010. In contrast, the number of cases and deaths due to lung diseases generally declined or was kept at a stable, low level in the USA and Australia, where the prevalence of CWP in Australia was still lower than 0.5%.
- The geological conditions of China, when compared with the USA and Australia, fundamentally disadvantages the country for coal mining; most coal mines need to be underground coal mines that compete with complex fault systems, and the coal rank and soft host rock increase dust production.
- Methods and standards for the mitigation of coal dust, including the main mining methods, engineering controls, and respiratory protective equipment, are weaker and less implemented in China compared with the USA and Australia, especially within the small coal mines. Meanwhile, the USA is the leading developer of new mitigation methods.
- A disparity in the occupational exposure limit existed among the three countries, where China and Australia have relatively low requirements compared to the USA. China was lagging behind the USA and Australia in terms of physical monitoring devices usage of respirable dust and inspection implements.
- The examination and diagnosis of lung diseases in China were less effective due to the lack of access to medical support such as mobile units or integrated health services compared with the USA and Australia. Likewise, there was a gap between the training of B readers/medical practitioners in the USA and China, which affects the precision of lung disease diagnoses. In addition, Australia has a more cooperative, integrated awareness of the examination and diagnoses of lung diseases than China.
- In China, the governance of occupational diseases occurred solely through the governments, which differed with the cooperative governance in the USA and Australia, where the government, industries, coal workers, unions, and so on had a higher degree of involvement and more effective issue resolution.
- Compared with the USA and Australia, the consequences for coal operators implicated in cases of occupational diseases were relatively lenient, and there is ineffective compensation for coal workers with lung diseases. Additionally, the health coverage for retired coal workers was not adequate in China.

Based on the above analysis and conclusions, the suggestions for Chinese coal workers’ lung diseases management are made as follows:

- To increase health awareness, keeping pace with safety management.

All levels of government, coal mines, and other stakeholders should put the health of coal workers at the same level of importance as safety or accident management. Meanwhile, the transfer of the Occupational Safety and Health Supervision Division from the NHFPC to the SAWS should be finalised as soon as possible, clarifying the departmental responsibilities and avoiding any existing overlaps or ambiguous definitions. In addition, the legislation on occupational health should be completed for all governments at all levels. For example, the penalty of health mismanagement such as concealing violations should be strengthened to match the level of current safety violation consequences.

- To accelerate technological control of mitigation of coal dust.

The automation of coal mines should be improved to reduce the number of coal workers who are exposed to coal dust on the work site. The safety and health department in China should research and review new global dust mitigation techniques and publish its findings to ensure that all those involved in Chinese coal mining are educated in the world-leading dust mitigation practices. Concurrently, the coal mine companies should put more importance on and increase investment in dust mitigation technology.

At the same time, the international and domestic cooperation should be enhanced to develop new technology for the mitigation of coal dust, taking full advantage of resources from the government, universities, and the coal industry.

- To update and popularise the monitoring devices of coal dust and perfect inspection management.

Firstly, the real-time personal dust monitors and respiratory protective equipment should be certified and popularised for use in underground coal mines as soon as possible. Secondly, the sample collection method should adopt personal sampling; therefore, the single-sample should be widely adopted. Likewise, the management system of statistics and re-detection, as well as the evaluation of the occupational exposure limit of coal dust, should be improved. In addition, the testing of the sample should be performed by a third party rather than the coal mine itself. Thirdly, unannounced inspections and mobile inspectors should be a regular part of mine operation, with increased inspection frequency. Similarly, the inspector should have the appropriate qualifications and knowledge to observe coal workers.

- To strengthen the health examinations for coal workers.

Firstly, the government should strengthen the construction and standardisation of occupational institutions and encourage cooperation with the NIOSH. Furthermore, medical practitioners in these institutions should be provided with educational resources and re-training to improve the accuracy rate for diagnoses of lung diseases. Secondly, multiple methods of examination should be implemented, for example, adopting the mobile unit model used by the NIOSH, which is capable of delivering chest X-rays, spirometry, and general health assessments for coal workers and former coal workers in regional mines. Thirdly, the frequency of health examinations for coal workers in hazardous regional areas should be improved. Fourthly, coal workers should be thoroughly educated in the respirable dust exposure risks and their rights regarding their health, either by the local government or their unions.

- To advocate cooperative governance for occupational diseases.

The government should advocate that social parties (unions, non-governmental organisations, and media campaigns), industry players, and all employers participate in the co-governance, that is, actively support dialogue and knowledge-sharing amongst all groups. The Occupational Safety and Health Supervision Division and local safety and health supervision departments should take over governmental duty and cooperate with other departments such as those related to finance, industry, information technology, civil affairs, and so on. Meanwhile, unions should become more independent and work towards coordinating and safeguarding the rights of coal workers; to implement this would mean improving the supervision and organisation within the unions themselves. In addition, advisory groups that have appropriate medical experience with lung diseases should be employed by the government to provide medical advice and promote cooperation between medical professionals and governing bodies. In China, most of the coal workers live in villages, so local village leaders and influential people should participate in co-governance.

- To reform regulation, simplify the process, and enhance the implementation of compensations for coal workers with lung diseases.

Firstly, the compensation standards for coal workers with lung diseases should be appropriately increased. Secondly, there should be clarifications on the responsibilities of the departments of labour, social security, and occupational diagnose institutions relating to the compensation process, as well as the simplification of that process without unnecessary bureaucracy. In conjuncture, the local departments of safety and health should play important roles in monitoring the compensation process. Likewise, the government ought to establish reporting and tracking of the progression of occupational diseases to the Occupational Safety and Health Supervision Division, where the claimants can report the implementation of benefits. Thirdly, the departments of labour ought to promote the implementation of labour contractors between coal workers and coal mines. Fourthly, compensations for coal works without labour contracts should be improved, and multiple compensation methods should be implemented, i.e., union, non-governmental, and rural cooperative medical system funds. Fifthly, rehabilitation and return to work programs should be established for those diagnosed with lung diseases in order to assist them to return to suitable alternative employment.

- To improve the health management of retired coal workers.

Ideally, there should be free health examinations for retired coal workers, not just an examination upon retirement, but regular examinations post-retirement. At the same time, the communication between retired coal workers and their governments and unions ought to assist in the evaluation, treating, counselling, and rehabilitation of retired coal miners with lung diseases. An example would be public information services through a toll-free telephone helpline or online service to give free and confidential advice to mine workers who have concerns about their respiratory health. Lastly, there should be compensation for retired coal workers, even those without retirement examinations.

- To establish a complete and efficient data system for the health assessment of occupational diseases

The health assessment system should involve all parties, including the government, occupational examination institutions, occupational diagnosis institutions, and the coal mines. Thereinto, digital radiography data storage in a single collaborative system provided by the occupational examination or diagnosis institutions can be accessible to workers, unions, government agencies, and employers. Another accessible system that should be provided is a comprehensive database of dust monitoring results as provided by the department of safety and health or coal mines. The government should identify the trends of diseases in mining, inform policy decisions, and identify regional areas or individual mines for potential scrutiny on the basis of the health assessment system.

Coal workers’ lung diseases have always been an important global public health concern. This paper strengthens the international understanding of coal workers’ lung diseases by systemic and comprehensive comparisons of management processes from the fundamental coal dust sources to the controllable treatment of coal workers’ lung diseases within China, the USA, and Australia. The systemic deficiencies of coal dust and health management practices in China compared with other countries provide important references for the improvement of these systems in China and other countries with serious occupational lung diseases. Additionally, this paper enriches the comparative systems of coal works’ lung disease, and extends the development of occupational management research. However, there are some limitations that require further research. This paper is a comparative review; thus, the quantitative analysis of coal dust exposure and other comparative indicators was not quite adequate. With the prevalence of complicated coal workers’ lung disease, future research is necessary to conduct a more accurate data survey and quantitative analysis with regard to redefining the coal dust and silica exposure limit based on national conditions and the clinical, pathological, and diagnostic studies based on different dust concentrations and coal ranks. Furthermore, further exploration into the mechanisms of cooperative governing involving the government, coal mines, workers, and social organisations is needed, including the judiciary, insurance, and healthcare aspects.

## Figures and Tables

**Table 1:** New cases of occupational diseases (pneumoconiosis, silicosis) for the total population and coal miners per year in China. CWP: coal workers’ pneumoconiosis.

Years	Occupational Diseases	Pneumoconiosis	Coal Workers’ Pneumoconiosis	Silicosis	Lung Disease of Coal Workers	The Proportion of CWP and Silicosis among Pneumoconiosis
2003	10,571	8361	4255	2836	4561	84.81%
2004	-	8743	-	-	-	-
2005	12,212	9173	4358	3967	4477	90.76%
2006	11,519	8783	3503	-	-	-
2007	14,296	10,963	5351	4447	6554	89.37%
2008	13,744	10,829	4924	4748	5471	89.32%
2009	18,128	14,495	7397	5922	7502	91.89%
2010	27,240	23,812	12,564	9870	13,968	94.21%
2011	29,879	26,401	14,000	11,122	-	95.16%
2012	27,420	24,206	12,405	10,592	13,399	95.01%
2013	26,393	23,152	13,955	8095	15,078	95.24%
2014	29,972	26,873	13,846	11,471	11,396	94.21%
2015	29,180	26,081	14,152	10,343	11,625	93.92%
2016	31,789	27,992	16,658	10,072	13,070	95.49%

Note: The data was primarily obtained from the Chinese Center for Disease Control and Prevention and National Health and Family Planning Commission [35,36,37,38,39,40,41,42,43,44,45,46], “-” represents the unavailable data.

**Table 2:** Comparisons of coal mine type, geological condition, mitigation, and monitoring of coal dust among China, the USA, and Australia. NIOSH: National Institute for Occupational Safety and Health, NSW: New South Wales, QLD: Queensland.

Category	Countries	China	The USA	Australia
Coal mine type (numbers)	Surface coal mine (*N*)	439 (in 2017)	1055 (in 2015)	NSW (22); QLD (37) (2017)
	Underground coal mine (*N)*	7223 (in 2017)	405 (in 2015)	NSW (20); QLD (13) (2017)
Geological condition	Structural geology	Complex geological structure Complex faulting and folding Soft rock, rock swelling Lean, thin, and medium coal seams	Slowly inclined or near horizontal coal seam Less structural damage such as faults, folds, and subsidence Medium and thick coal seams	Slowly inclined or near horizontal coal seam Medium and thick coal seams
	Rank of reserved coal	Lignite (13%) All kinds of bituminous coal (75%) Anthracite (12%) (in 2017)	Bituminous (44.40%) Sub-bituminous (45.33%) Lignite (10.04%) Anthracite (0.23%)	Black coal (48.56%) Brown coal (51.44%)
Mitigation of coal dust	Main mining methods	Conventional mining (Small coal mines) Longwall mining method (single, top coal caving, inclined stratification)	Opencast mining (draglines and truck and shovel operations, or a combination of the two) Longwall mining method, room pillar	Opencast mining (draglines and truck and shovel operations, or a combination of the two) Longwall mining method, room pillar, and punch longwall mining
	Main engineering control methods	Ventilation Coal seam water infusion Water sprays (dust curtains, shield canopy sprays, ranging arm sprays, etc.) and other wetting agents (relatively single) Blisters mud and foam dust control (surfactant) Automation and remote equipment operation	Watering trucks Ventilation Coal seam water infusion Water sprays (dust curtains, shield canopy sprays, ranging arm sprays, etc.) and other wetting agents with multi-chemical application i.e., calcium, magnesium chloride, sodium silicate, etc. Foam dust control (surfactant) Automation and remote equipment operation New dust removal technology (ultrasonic dust removal, biological reagent deducting, etc.)	Watering trucks Ventilation Coal seam water infusion Water sprays (dust curtains, shield canopy sprays, ranging arm sprays, etc.) and other wetting agents (relatively single) Foam dust control (surfactant) Automation and remote equipment operation
	Respiratory protective equipment	Particulate respirators: (GB2626-2006) Nonpowered particulate respirators Powered air-purifying respirators, negative pressure respirators, including KN and KP (KN 90KN95KN100; KP 90KP95KP100) Common type of coal miners: KN 95 and half mask	Particulate respirators: (42 CFR Part 84) Nonpowered particulate respirators Powered air-purifying respirators, negative pressure respirators, including N, R, P (N95N99N100; R95R99R100; P95P99P100) high-efficiency particulate air (HEPA) filter, including N100, R100, and P100 filters Common type of coal miners: N95 (or higher) filters and half mask (NIOSH-recommended related respiratory protection for workers exposed to different respirable coal mine dust and respirable crystalline silica)	Particulate respirators: (AS/NZS 1715 and 1716) Nonpowered particulate respirators Powered air-purifying respirators Per AS/NZS 1715, there are three different classes of particulate filters, P1, P2, and P3 (P1: Intended for use against mechanically generated particulates, for example, silica, asbestos, etc.) Common type of coal miners: P1 and half mask
Monitoring of coal dust	Occupational exposure limit	The concentration of free silica (CFS): <5%, the respiratory dust limit (RDL): 5.0 mg/m^3^ CFS: 5–10%, RDL: 2.5 mg/m^3^ CFS: 10–30%, RDL: 1 mg/m^3^ CFS: 30–50%, RDL: 0.5 mg/m^3^ CFS: ≥50%, RDL: 0.2 mg/m^3^ (AQ 4202-2008) (TWA)	Before 2014, RDL: 2.0 mg/m^3^ (CFS < 5%) RDL free silica: 0.1 mg/m^3^ (CFS >5%) (American Conference of Governmental Industrial Hygienists (ACGIH) and Occupational Safety and Health Administration (OSHA), TWA) Since 2014, RDL: 1.5 mg/m^3^ (CFS < 5%); free silica: 0.1 mg/m^3^ (CFS > 5%) [Mine Safety and Health Administration (MSHA), TWA] In 2016. RDL: 1.0 mg/m^3^ to 0.5 mg/m^3^ for intake air at underground mines and for ‘part 90’ miners (coal miners who have evidence of the development of pneumoconiosis)	Two main states of a coal mine (TWA) QLD (Queensland) coal dust concentrations: 3 mg/m^3^ free silica: 0.1 mg/m^3^ NSW (New South Wales) coal dust concentrations: 2.5 mg/m^3^ free silica: 0.1 mg/m^3^
	Monitoring devices	Traditional gravimetric sampling device Static monitoring devices Real-time personal monitoring devices: (light scattering (laser photometry) devices	Traditional gravimetric sampling device Static monitoring devices Real-time personal dust monitoring devices: light scattering (laser photometry) devices and tapered element oscillating microbalance (TEOM) devices (more)	Traditional gravimetric sampling device Static monitoring Real-time personal dust monitoring devices: light scattering (laser photometry) devices (more) and tapered element oscillating microbalance (TEOM) devices
	Common sampling methods	Area sampling (Many) Personal sampling (8 h) Respiratory sampling	Area sampling (Less) Breathing zone (Less) Designated operator sampling	Area sampling (Less) Personal sampling (8 h and in their breathing zone for an extended period of their work shift)

Note: Black coal includes anthracite, bituminous coal, and sub-bituminous coal; Brown coal includes lignite (coal classification terminology in Australia); TWA: Threshold Limit Value.

**Table 3:** The comparison of various health assessment factors among China, the USA, and Australia.

Category	China	The USA	Australia
Periodic health examination	Health examination (spirometric examination and chest radiograph) every two to three years Health examination at the end of employment in coal mining Different periods depend on coal mine companies	Spirometric examination each year for the first three years of coal mining, every two to three years after that if the miner remains engaged in coal mining A chest radiograph every four to five years for the first 15 years of coal mining and every three years after that if the miner remains engaged in coal mining A chest radiograph and spirometric examination at the end of employment in coal mining if more than six months have passed since the last examination.	QLD: Health examinations every five years for underground coal workers, and every 10 years for surface coal workers NSW: A chest X-ray is required every six years if the worker has a high risk of dust exposure in open-cut or underground minutes; after 2017, increasing the frequency of chest X-rays to three years for underground coal miners and at risk surface coal miners, with a maximum period of six years for all other coal miners. Health examination at the end of employment in coal mining
Participants	Coal mine company (coal mine operators) Government (National Health and Family Planning Commission (NHFPC), State Administration of Work Safety (SAWS), Occupational Safety and Health Supervision Division (OSHSD), etc.) Coal mine workers Unions and non-governmental organisation (China Occupational Safety and Health Association (COSHA), China Coal Miner Pneumoconiosis Treatment Foundation (CMPTF), Love Save Pneumoconiosis (LSP))	Coal mine company (coal mine operators) Government (Occupational Safety and Health Administration (OSHA), Mine Safety and Health Administration (MSHA), National Institute for Occupational Safety and Health (NIOSH), etc.) Union and non-governmental organisations (United Mine Workers of America (UMWA), American Lung Association (ALA), Centers for Disease Control and Prevention (CDC), etc.) Coal mine workers Community	Coal mine company Government (Coal Mining Safety and Health Advisory Committee (CMSHAC), health surveillance unit (HSU), Department of Natural Resources and Mines (DNRM), etc.) Union (Construction, Forestry, Mining, and Energy Union (CFMEU), Australian Manufacturing Workers’ Union (AMWU)), etc.) Coal mine workers Community
Pattern of governance	Government-dominated governance	Co-governance	Co-governance
Standard of radiograph	Chinese National Diagnosis Criteria of Pneumoconiosis (GBZ 70-2009)	International Labour Office (ILO) International Classification of Radiographs of Pneumoconiosis	International Labour Office (ILO) International Classification of Radiographs of Pneumoconiosis
Insurance	WorkCover Funds of non-governmental organisations	WorkCover Self-insurer Funds such as the health and retirement funds of UMWA Funds of non-governmental organisations	WorkCover Self-insurer Mining safety fund (NSW) and victim funds of industry levy (QLD) Funds of non-governmental organisations
Compensation	A fee of medical treatment and rehabilitation Disability subvention standard of WorkCover: 10 to 1 rank, pay a lump sum payment of six to 24 months’ salary depending on the different ranks Disability benefits standard of WorkCover: 6 to 1 level, pay 60–90% of monthly salary until the retired age. The 1, 2, 3 of CWP was generally evaluated as the level 7, level 4, and level 2 of occupational injuries (Retired workers could enjoy the benefits of the basic old-age insurance pension after retirement, stopping the WorkCover)	Part B, Black lung monthly benefit rates paid by the department of labour from 660.10 USD to 1320.1 USD based on the number of dependents Part C, Black lung monthly benefit rates paid by social security administration from 660.10 USD to 1320.1 USD based on the number of dependents Compensation of states (Division of Coal Mine Workers’ Compensation (DCMWC)) If a miner or surviving spouse is receiving workers’ compensation, unemployment compensation, or disability insurance payments under state law, the Black lung benefit is offset by the amount being paid under these other programs In addition to compensation, miners with occupational lung disease that interferes with work or causes disability may be eligible for rehabilitation and medical treatment or retraining	Basic expenses of cure: reasonable hospital, medical, and ancillary expenses (e.g., treatment, medicines, ambulance, etc.), occupational rehabilitation services; other reasonable expenses incurred while seeking medical treatment, such as travel expenses Worker’s injury is stable, and a lump sum payment has been accepted based on permanent impairment; the maximum compensation payable for permanent impairment was increased to a maximum of 577,050 AUD for workers with a permanent impairment of 75% or more (NSW) Weekly benefits during any period of incapacity received to reach the maximum amount (325.70 AUD as of 1 April 2018) (NSW) The worker has received weekly payments of compensation for five years
Retiring coal mine workers	Free retirement examination (Voluntary) Free health assessment coving the retired worker (only in state-owned and formal labor contract)	Free retirement examination Free health assessment coving the retired worker (Voluntary) A network of clinics offers specific experience and resources in evaluating, treating, counseling, and rehabilitating	Free retirement examination Retired and former mine workers have access to health assessments after their employment has ended (since January 2017 in QLD) (Voluntary) Union communicated the resources in evaluating, treating, counseling, and rehabilitating
Penalty of Violation	If coal mines conceal the occupational diseases, punish the coal mines fine of 10,000 to 50,000 RMB, warning or order date of expiration correction, or demotion or dismissal of operators	Operators who knowingly conceal or dispose of any property to avoid the payment of benefits under the Act may be guilty of a misdemeanour and, if convicted, subject to a fine of $1000, imprisonment for up to one year, or both.	The penalty for the discharge of obligations for operators involving exposure to a harmful substance (update 2018): fine of 750 penalty units, to be consistent with the existing maximum penalty unit amount 126,000 AUD and have right of prosecuting an operator or shut down a mine or suspend or cancel statutory certificates of competency (QLD)
Health assessment methods	Chest radiograph (X-ray) Respiratory assessment questionnaire Spirometry testing	Chest radiograph (X-ray) Respiratory assessment questionnaire Spirometry testing Mobile units	Chest radiograph (X-ray) Respiratory assessment questionnaire Spirometry testing Mobile units (only in NSW)
